# Bioaccumulation and Biomagnification of Hexabromocyclododecane in Marine Biota from China: A Review

**DOI:** 10.3390/toxics10100620

**Published:** 2022-10-19

**Authors:** Ying Zhang, Sijia Li, Yafeng Zhang, Yezi Chen, Xutao Wang, Yuxin Sun

**Affiliations:** 1 Eco-Environmental Monitoring and Research Center, Pearl River Valley and South China Sea Ecology and Environment Administration, Ministry of Ecology and Environment of the People’s Republic of China, Guangzhou 510611, China; 2 School of Marine Sciences, Sun Yat-sen University, Guangzhou 510006, China; 3 Southern Marine Science and Engineering Guangdong Laboratory (Zhuhai), Zhuhai 519000, China; 4 Guangdong Provincial Key Laboratory of Chemical Pollution and Environmental Safety, School of Environment, South China Normal University, Guangzhou 510006, China

**Keywords:** HBCD, marine organisms, bioaccumulation, biomagnification

## Abstract

Hexabromocyclododecane (HBCD) was listed in Annex A of the Stockholm Convention on Persistent Organic Pollutants for its persistence, bioaccumulation and toxicity, and pose significant adverse effects on natural environments and human health. HBCDs are ubiquitously found in marine environments worldwide and can be biomagnified in marine organisms with a high trophic level. In the present study, we reviewed the available data on contamination of HBCDs in the marine biota from China, including mollusks, crustaceans, fish and mammals. Bioaccumulation and biomagnification of HBCDs in the marine food web were summarized as well. This study also prospected the future research of HBCDs, including the transport and fluxes of HBCDs to and within the marine environment, the biomagnification of HBCDs in different ecosystems, and the metabolism of HBCDs in different marine species.

## 1. Introduction

Hexabromocyclododecanes (HBCDs) were the third most used brominated flame retardants (BFRs) worldwide after polybrominated diphenyl ethers (PBDEs) and tetrabromobisphenol A (TBBPA). HBCDs have been widely used in buildings, textiles and electronics as a substitute for PBDEs due to their lower dosage, better effect and smaller impact on materials [1]. HBCDs are typical hydrophobic flame retardants with high bromine content (log*K*ow = 5.4–5.8), which can enter the environment by a number of different pathways, such as emission during the production process, by leaching from consumer products, or following disposal. HBCDs have been found in practically all environmental media including air, water, sediment, biota, human blood and milk [2,3,4,5,6], and their biomagnification along the food chain was investigated [7,8,9,10].

HBCDs can cause adverse effects to the thyroid and liver, increase cholesterol and tibial bone mineral density, inhibit egg development and neural development in rats [11], and in severe cases, liver necrosis and cancer may occur in rats exposed to HBCDs [12]. Chronic toxicity of HBCDs was also shown in aquatic life although its acute toxicity is low [13], such as the significant toxic effects on the reproductive development of marine invertebrates [14]: the malformation rate increased and survival reduced significantly in HBCD exposed zebrafish [15,16,17]. Thus, HBCDs may be harmful to humans through the process of biomagnification.

In May 2013, HBCDs were listed in Annex A of the Stockholm Convention on Persistent Organic Pollutants. In December 2016, the production, use, import and export of HBCDs were prohibited in China. In December 2017, HBCDs were listed in the first batch of the list of Priority Chemicals under Control in China. However, because it is hard to degrade, HBCDs still exist in the environment worldwide, and can enter the ocean by rivers, surface runoff and sewage outlets.

The ocean is valuable in the process of sustainable development, and it is of great significance to the survival and development of mankind. The marine economy has become one of the most dynamic and promising areas for economic growth in coastal countries. At the same time, increasing concern is also focused on marine pollution. This study reviewed the bioaccumulation and biomagnification of hexabromocyclododecane in marine organisms in China based on the latest research studies, in order to provide reference for the study of HBCDs in marine biota.

## 2. Bioaccumulation of HBCDs in Marine Biotas

As will be discussed in detail further below, HBCDs were generally detected in various marine organisms along the Chinese coastline (Table 1).

*Mollusks***.** Concentrations of HBCDs in mollusks were mainly reported from Bohai sea and Honk Kong. The mean value of the total HBCDs in mollusks ranged from 25.1 to 517 ng/g lw around the Bohai sea, China [18,20], while the concentrations in mollusks from Hong Kong waters were lower (13.7 to 29.1 ng/g lw) than those around the Bohai sea [10]. The relatively higher levels of HBCDs around the Bohai sea are possibly related to human activities, since serious pollution of Bohai Bay waters by domestic and industrial effluents has occurred [26]. In comparison with other countries, HBCD concentrations in mollusks from China were comparable to those from South Korea [27] and Europe [28], while 2–3 times higher than those from the Californian coast, and about 100 times lower than the highest concentration in Japan (5200 ng/g lw) [29].

**Figure 1:**
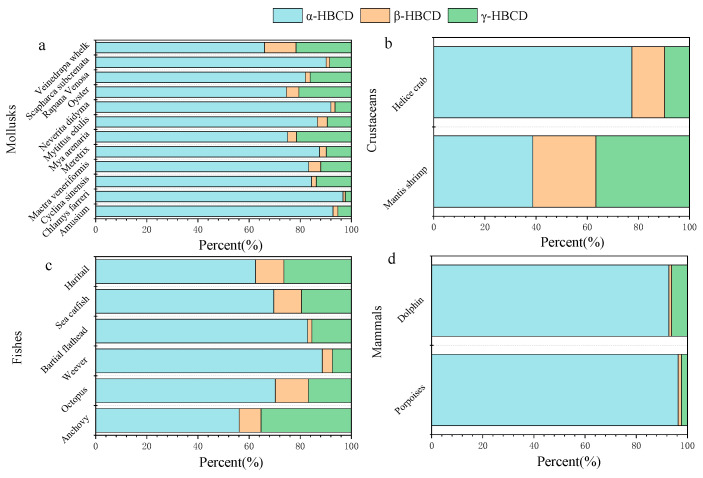
Diastereoisomer patterns of HBCDs in marine biota from China. (**a**) mollusks; (**b**) crustaceans; (**c**) fishes; (**d**) mammals.

The diastereoisomeric pattern of HBCDs in mollusks from the Bohai sea were as follows: α-HBCD (66.0–96.8%) > γ-HBCD (2.4–21.7%) > β-HBCD (0.9–12.3%)(Figure 1a).γ-HBCD contributes more than 80% to the total HBCDs in commercial mixtures, and in general, γ-HBCD was detected mainly in environmental media such as air, surface soil and sediments [6]. The dominance of α-HBCD has been also observed in mollusks from other countries [27,29]. This pattern indicated a higher bioaccumulative potential of α-HBCD and/or higher metabolism of γ-HBCD and β-HBCD. γ-HBCD and β-HBCD were metabolized more quickly than α-HBCD, resulting in an enrichment of the α-HBCD. Haukås et al. (2010) have found that ragworm selectively bioaccumulated α-HBCD from mussels [30], and transformations from γ-HBCD or β-HBCD to α-HBCD were observed in aquatic organisms in the laboratory [31,32].

The molecular response of the gills of clams exposed to HBCDs at environmentally relevant exposure levels was investigated and significant differentially expressed genes involved in stress, defense response and metabolism were observed [33]. However, toxicity and the molecular mode of HBCDs in mollusks were rarely reported, further studies are necessary to understand the response and adaptation mechanisms of these organisms under multiple environmental stressors.

*Crustaceans.* The concentrations of the total HBCDs in shrimps and crabs were in the range of 39.5–250 ng/g lw and 48.0–428 ng/g lw, respectively [19,20,21]. The levels of HBCDs in crustaceans from Hong Kong (4.83–58.6 ng/g lw) were lower than those from Tianjin, which was similar to the situation with mollusks. The concentrations of ƩHBCDs in crustaceans from China were comparable to those (38–46 ng/g lw) from the Western Scheldt Estuary [34], and much higher than those from Japanese coastal areas [35]. 

The diastereoisomeric pattern of HBCDs in crustaceans from Tianjin is shown in Figure 1b. In the study of crustaceans collected from the mouth of the Haihe River to the Bohai Sea, the similar percentage between α-HBCD (38.7%) and γ-HBCD (36.6%) was found in mantis shrimp, while α-HBCD was the predominant isomer (77.5%) in helice crab [20]. The higher percentage of γ-HBCD (49.5%) than α-HBCD (32.8%) was found in crustaceans collected from Hong Kong waters [10]. The similar result was also found in crustaceans from estuaries of the Mihe River to the Bohai Sea [19]. However, α-HBCD was the predominant isomer in both, shrimps and crabs from Tianjin coast [21].The higher percentage of γ-HBCD (about 40%) in shrimp than those in other aquatic organisms were also found in Western Scheldt Estuary [34]. 

Relatively few studies evaluated the toxicity of HBCDs on marine crustaceans. HBCDs were found to affect the ingestion and filtration rates of the marine copepod in a short-term sub-lethal exposure experiment [36]. Marine copepod exposed to HBCDs caused significant growth delay, particularly during the nauplius phase [37]. As a key link of marine organic matters migrating from primary production to higher trophic levels, the changes on ingestion capacity or metabolic level of invertebrates could have a substantial effect on the energy flow and circulation of material in marine ecosystems. Therefore, more studies on the toxicity of HBCDs in invertebrates should be analyzed to assess the impacts of HBCDs on the entire marine primary ecosystem [38].

*Fish.* HBCDs were widely detected in fish from Chinese coastal areas, including Tianjin and Dalian in northern China, the coastal areas of eastern China and the Pearl River Estuary in southern China [10,20,22], with the mean concentration ranging from not detected to 2970 ng/g lw. Xia et al. (2011) investigated HBCDs in two fish species from nine Chinese coastal cities, and found the relatively higher levels of HBCDs in pomfrets from the cities on the coasts of the Bohai and Yellow Seas than those from the cities on the coast of the East China Sea [22]. Much higher levels of HBCDs in fish from Tianjin (5.2–2970 ng/g lw) than those from southern coastal of China (nd-93.23 ng/g lw) were also observed in other studies [10,21,22,23,25]. The concentrations of ƩHBCDs in fish from China were comparable to those (nd-1113 ng/g lw) from the Western Scheldt Estuary, and much higher than those (nd-4.64 ng/g lw) from the European markets [28].

Similar to the mollusks, the percentage of α-HBCD (56.1–88.6%) > γ-HBCD (7.4–35.4%) > β-HBCD (1.7–12.9%) were also seen in marine fish from Tianjin coastal (Figure 1c) [20], and the similar compositions were also found in marine fish from other coastal areas of China [19,21,23]. The selective absorption and metabolism of HBCD and isomerization in organisms may result in the isomeric bioaccumulation. Law et al. (2006) firstly reported the transformation of β-HBCD and γ-HBCD to α-HBCD in juvenile rainbow trout (*Oncorhynchus mykiss*), and α-HBCD was detected in zebrafish exposed to γ-HBCD at day 28 of the uptake phase [31]. 

HBCDs can cause adverse effects on the survival of marine fish. HBCDs were demonstrated to significantly increase the malformation rate and reduced survival for zebrafish embryos at relatively high exposure concentration (up to 1 mg/L). HBCDs induce the generation of reactive oxygen species (ROS) and caspase-3 and -9 mediated apoptosis [16,17]. Marine medaka exposure to HBCDs at environmentally realistic concentrations resulted in development toxicity, including induced oxidative stress and apoptosis, and suppressed nucleotide and protein synthesis, particularly in the cardiovascular system [39].

*Mammals.* HBCDs were only reported in finless porpoises and Indo-Pacific humpback dolphin from Hong Kong waters with concentrations in the range of 4.1–6260 ng/g lw and 97.2–45580 ng/g lw, respectively. The levels of ƩHBCDs in marine mammals in China were higher than those in Southern European, Indian, Eastern and Western United States coasts [40,41,42,43,44,45], and were similar to those from the UK coast [46].

Lam et al. (2009) found that α- HBCD contributed 96.4% and 92.8% to ƩHBCDs in porpoises and dolphins (Figure 1d), respectively [24]. The predominance of α-HBCD in mammals among the studied physiological traits was also found in the study of Ruan et al. [25]. The occupancy at the top of the marine food web may explain that α-HBCD usually had an absolute predominance or was even the exclusive diastereomer detected in marine mammals. Except the three common isomers (α-, β-, γ-HBCD), δ- and ε-HBCD were also found in porpoises and dolphins, which may reflect a combined consequence of bioaccumulation and biotransformation (including bioisomerization). There are very limited number of studies addressing the δ- and ε-meso isomers, probably because there are very few records on the two isomers in environmental matrices [47].

As the top predator, mammals (including humans) can be exposed to HBCDs through dietary intake. Diet is considered one of the major pathways of exposure [47], especially through seafood [48,49]. Based on the toxicities of HBCDs, humans are expected to be adversely affected by ingesting food contaminated with HBCDs. Fery et al. (2010) identified HBCD as a common activator of the human PXR in Hep G2 cells [50]. Cytotoxicity of HBCDs in human Hep G2 cells were observed at relative lower doses (0.5 and 1 μg/mL) which can frequently be detected in biotic and environmental samples [51]. The human health risk from dietary exposure of HBCD can be assessed using a hazardous quotient (HQ) approach (HQ = EDI/reference dose), when HQ < 1 is no obvious health risk, HQ ≥ 1 suggest obvious health risk to the exposed population. US National Research Council proposed an oral reference dose (RfD) of HBCD for humans with the value of 2 × 10^5^ ng/kg/day while the European Food Safety Authority (EFSA) proposed a chronic reference dose for HBCD of 7.9 × 10^5^ ng/kg/day. According to the study on the human exposure to HBCDs in a coastal ecosystem near a large manufacturer in China, the total EDI from the fish and shellfish was 5.22 ng/kg/day for adults while it was 16.39 ng/kg/day for children [19], but the EDI from aquatic food for general population was only 0.08 ng/kg/day in China [52]. Although EDI indicated a very low risk of HBCD from the aquatic food consumption, limitations should be noted, as the RfD may change when more toxicity studies become available, and that local population can intake HBCD through other food or other pathways such as inhalation and occupational exposure.

The bioconcentration/bioaccumulation factor (BCF/BAF) and biota-sediment accumulation factor (BSAF) are used frequently to evaluate the toxicity of pollutants in aquatic organisms, and to develop environmental standards and guidelines [53]. BCF/BAF > 5000 (log BCF/BAF > 3.7) indicates that aquatic biotas can enrich pollutants from water and BSAF > 1 indicates that pollutants in sediments can be enriched by organisms. In freshwater ecosystems, the average field-derived BAFs for 12 fish species collected from 9 lakes in the UK were 5900, 1300, 810, and 2100 for α-, β-, γ-, and ƩHBCDs, respectively [54]. The log BAFs for aquatic organisms collected from an e-waste recycling site in South China were 2.58–6.01, 3.24–5.58, 3.44–5.98, and 2.85–5.98 for α-, β-, γ-, and ƩHBCD, respectively [55]. Wang et al. (2018) have studied the tissue-specific BSAF values for crucian carp collected from river basins in South Korea, and found that liver samples had the highest BSAF value (19357), followed by egg (19119), blood (2413), and muscle (1504) [56]. However, higher BCFs/BAFs of HBCD were observed in the marine environment, for example, BCFs > 41,700 for HBCDs were found in oyster and mussel collected at sites in four bays and Gal Island in South Korea [27], and log BAFs > 5.5 were found in oysters collected from the coastal areas of Okinawa, Japan [57]. In the present study, HBCDs had BCFs/BAFs greater than 3.7 (corresponding BAF value 5000) in most of the investigated aquatic species, demonstrating HBCDs are highly bioaccumulation chemicals, and the different species and aquatic environment may account for the various BCFs/BAFs between different regions.

In comparison with BCF/BAF, little data on BSAF were available. BSAFs < 1 of HBCDs were observed in invertebrates collected from South Korea (mean values of 0.02 for oysters and 0.04 for mussels) [27] and Japan (0.01–0.89 for oysters) [57], but higher BSAFs of HBCDs (0.10–1.44 for bleak and 0.14–1.23 for barbell) were seen based on an uptake model [58]. For the tissue-specific bioaccumulation, the highest BSAF value in crucian carp tissue was found in liver (25.62), followed by egg (11.63), blood (2.83), and muscle (1.11) [56]. Due to the extensive existence of HBCDs and their individual toxicities, more research should be conducted to increase our understanding of HBCDs bioaccumulation especially for marine biotas.

## 3. Biomagnification of HBCDs in Marine Food Web

Biomagnification factor (BMF) and trophic magnification factor (TMF) were commonly used to investigate the biological magnification behavior of pollutants in the food chain. BMF was defined as the ratio of the average lipid-normalized concentration between predator and prey [59]. TMF was used to describe the biomagnification, and the calculation was based on the correlations between average lipid-normalized concentration and trophic levels (TLs) [60]. BMF and TMF values > 1 indicate biomagnification, while values between zero and one imply that the compound is present throughout the food web but is not being biomagnified.

Several studies have investigated the biomagnification properties of HBCDs in the marine ecosystem of Chinese coast (Table 2). Zhang et al. (2013) studied in the mouth of the Haihe River to the Bohai Sea, by analyzing mantis shrimp, veinedrapa whelk, helice crab, anchovy, octopus, weever, bartial flathead, sea catfish and hairtail for HBCDs. The concentrations of α-HBCD and the total HBCDs increased with trophic level (*p* < 0.05), with TMF values of 1.74 and 1.68, respectively, indicating biomagnification throughout the food web. β-HBCD and γ-HBCD did not show any statistically significant correlation between the concentrations and the TLs (*p* > 0.05). The different metabolic behavior of β-HBCD and γ-HBCD in the web and the low concentrations in the organisms compared to α-HBCD might explain the lack of correlation between the concentrations and the TLs [20].

Mantis shrimp, helice crab, and bartial flathead were collected from eight locations along Tianjin coast to investigate the environmental fate of HBCDs. Concentrations of total HBCDs and three HBCD diastereoisomers increasing with TL were observed with TMF of 1.75, 1.83, 1.64 and 1.74 for α-, β-, γ-, and total HBCDs, respectively [21]. Their values are almost the same as the results of the previous study (1.74 for α-HBCD and 1.68 for total HBCDs, respectively) in the same area [20].

Moreover, a total of 161 aquatic specimens of 12 species were collected from the estuary of Mihe River to the Bohai Sea to illustrate the trophic transfer of HBCDs, including clam, oyster, shrimp, mantis shrimp, conch, crab, mullet, flathead fish, tongue sole, perch, porgy and goby. Among these species, there were 11 pairs of prey-predator relations, and the BMF values ranged from 0.9 to 28.1 for the 11 predator-prey pairs, suggesting biomagnification of HBCDs through these food chains. In this study, α-HBCD showed significant (*p* < 0.05) positive association with trophic level, while β-HBCD and γ-HBCD did not. The TMF value of α-HBCD was 10.8, indicating that the concentration of α-HBCD increased substantially with the increasing trophic level. Due to the predominance of α-HBCD in biota, the total HBCDs also showed positive correlation with trophic level (*p* < 0.05), with a TMF of 5.6, which may lead to even higher level of HBCD in top predator, such as human being [19]. However, in the study of a mangrove ecosystem from Jiulong River Estaury, negative but not significant correlations were found for α-HBCD with a TMF of 0.17 (*p* = 0.27), indicating no biomagnification of α-HBCD in the studied mangrove food web, which was consist of five species, including striped mullet, mud crab, red eelgoby, Chinese black sleeper and blue-spotted mudskipper. 

The biomagnification of HBCDs was studied in the marine food web from the western, southern, and eastern Hong Kong waters, including 5 mollusk species, 6 crustacean species, 19 fish species and 2 cetacean species [10]. The BMFs of ƩHBCD ranged from 15.0 to 192 for the predator-prey pairs of porpoises, and much higher BMFs were observed for the predator-prey pairs of dolphins, with values ranging from 325 to 4160 for ƩHBCD. In accordance with the predominance of α-HBCD in cetacean species, the BMFs of α-HBCD were the largest (16.8–7223). Although the levels of β-HBCD in marine mammals were detected relatively lower than those of γ-HBCD, higher biomagnification potential happened in β-HBCD, with the BMFs of 3.06–524 and 1.56–45.9 for β-HBCD and γ-HBCD, respectively, possibly because β-HBCD is present in relatively small quantities in technical products and is susceptible to biotransformation [32,65]. The increasing of ƩHBCDs and α-HBCD concentrations with the TLs were significant, with the TMFs of 7.9 and 10.3, respectively, indicating the strong biomagnification of ƩHBCDs and α-HBCD in the food web. There were no significant correlations in the concentrations of β–HBCD and γ-HBCD with the TLs.

Reports on BMFs and TMFs for HBCDs especially in marine ecosystems from other countries are scarce. Trophic magnification was observed for ƩHBCD, α-HBCD and γ-HBCD in marine food web from the southern part of Korea, with the reported TMFs higher than 1, while trophic dilution (TMF < 1) through the food web was found for β-HBCD [9]. Reindl and Falkowska (2015) investigated the BMFs of HBCD for the predator-prey pairs of African penguins-Baltic herring from the Gdansk Zoo, and found biomagnification for HBCD, with the BMFs ranging from 5.6 to 8.3 [61]. In the Western Norwegian coastal food web, the BMFs for α-HBCD ranged from 2.8 to 26 in the great black-backed gull predator-prey relationships. The TMF of α-HBCD was 2.6, indicating biomagnification of this diastereomer while both TMF and BMFs for γ-HBCD were below 1 [61]. Tomy et al. (2008 and 2009) studied the biomagnification of HBCDs in marine food web from the Western and Eastern Canadian Arctic, and the trophic magnification of α-HBCD and the trophic dilution of γ-HBCD were observed in the Eastern Canadian Arctic [63,64]. In the marine ecosystem on King George Island, Antarctica, the TMFs higher than 1 were generally observed for ƩHBCD, α-, β-and γ-HBCD, indicating that the levels of HBCDs were magnified through the food web.

The biomagnification of HBCDs through the marine food web in the mouth of the Haihe River to the Bohai Sea were comparable to those of Korea, Western Norway and Canadian Arctic while the TMFs of HBCDs in the marine food web from the estuary of the Mihe River to the Bohai Sea and Hong Kong waters were much higher than those in other countries. The different food web structures, wildlife habitats and biological metabolism may result in the differences of TMFs.

## 4. Summary and Future Perspectives

Although the production and application have been already banned, HBCDs are still commonly detected in the environment for their persistence. As the marine environment is an important destination for pollutants, it is of great significance to study the process of absorption, accumulation, transformation and transmission influencing factors of HBCDs in marine organisms for understanding the migration behavior of HBCDs and evaluating the risk of HBCDs pollution in marine ecosystems. Bioaccumulation of HBCDs were prevalently investigated in marine mollusks, crustaceans, fishes and mammals from China, especially for α-HBCD, due to their persistence, lower volatility and high solubility. Local usage and environmental behaviors of HBCDs, as well as discrepancies in habitat, diet and metabolism in different species, likely play important roles in the occurrence and bioaccumulation of HBCDs in marine organisms. Trophic biomagnification of ƩHBCDs and α-HBCD were observed in almost all the marine food web, while the trophic-dilution of β-and γ-HBCD were investigated in some studies Many factors, such as food web structures, diet diversity and physiological metabolism, may result in the differences of TMFs.

To better understand and manage the potential environmental risks associated with HBCDs, the following areas of research should be prioritized. First, the transport and fluxes of HBCDs to and within the marine environment must be further investigated. The pollution of HBCDs in China is more complex than that in the developed countries, such as Europe and the United States. In addition to entering the environment by the process of the local industrial production, the pollution of HBCDs caused by the transfer of global industrial chain (such as the export of electronic waste, chemical industries and low-end manufacturing industries) is also important. China has become the world’s largest manufacturing base and e-waste recycling base. The land-sourced pollutant can enter the marine by various ways, such as surface runoff, sewage outlet and atmospheric precipitation, likely to cause the threat to marine organisms. Therefore, studying on the flux of HBCDs into the sea is conducive to evaluate the potential ecological risks of HBCDs to marine ecosystems.

Future research will be needed to understand the biomagnification of HBCDs in different ecosystems. Compared with the marine environment, the environmental exposure pathways of HBCDs and the food web structures in the terrestrial environment are more complicated and varied. The biological diversities are varied between different geographic zones of climate. Borgå et al. (2012) have suggested that the food web structure should consist of three trophic levels of organisms at least and include homothermal animal when studying the biomagnification of persistent halogenated compounds [66]. At the same time, it is more difficult to construct terrestrial food webs in subtropical and tropical areas where the organisms are much more diverse and complex. More research should be conducted to reveal the transmission process of HBCDs in subtropical and tropical regions.

Finally, the metabolisms of HBCDs in organisms are poorly understood, while it is important to understand the metabolism efficiency of HBCDs in biotas and its effect to bioaccumulation processes before studying the biomagnification of HBCDs in the food web. There are many species in the food web, and the metabolism of HBCDs differed among biological species. Current research methods are difficult to quantitatively characterize the biological metabolism of pollutants. In the future, more advanced geochemical methods, such as compound-specific carbon isotopes, should be introduced to accurately quantify the effects of biological metabolism and food intake on HBCDs bioamplification.

## Figures and Tables

**Table 1:** Concentrations of HBCDs in marine organisms from China (ng/g lipid weight).

Location	Sample Time	Species	Concentration Range	Mean	Reference
		*Mollusks*			
Bohai sea	2009–2010	Amusium	7.69–59.2	34.2	[18]
Bohai sea	2009–2010	Chlamys farreri	9.93–147	48.2	[18]
Bohai sea	2009–2010	Cyclina sinensis	4.40–98.8	35.8	[18]
Bohai sea	2009–2010	Mactra veneriformis	nd–370	148	[18]
Bohai sea	2009–2010	Meretrix	nd–103	25.1	[18]
Bohai sea	2009–2010	Mya arenaria	25.4–134	80.2	[18]
Bohai sea	2009–2010	Mytittus edulis	23.37–166	78.9	[18]
Bohai sea	2009–2010	Neverita didyma	6.62–151	47.8	[18]
Bohai sea	2009–2010	Oyster	12.2–129	58.9	[18]
Bohai sea	2009–2010	Rapana Venosa	4.20–162	49.2	[18]
Bohai sea	2009–2010	Scapharca subcrenata	3.03–119	39	[18]
Tianjin	2015	Oyster		517	[19]
Tianjin	2015	Conch		367	[19]
Tianjin	2015	Clam		44.3	[19]
Tianjin	2011	Veinedrapa whelk		184.7	[20]
Hong Kong	2012	Anadara ferruginea		14.95 ± 4.51	[10]
Hong Kong	2012	Corbula crassa		14.30 ± 4.01	[10]
Hong Kong	2012	Turritella bacillum		15.2 ± 1.00	[10]
Hong Kong	2012	Murex trapa		29.13 ± 6.33	[10]
Hong Kong	2012	Bufonaria rana		13.72 ± 3.70	[10]
		*Crustaceans*			
Tianjin	2011	Mantis shrimp		138.97	[20]
Tianjin	2011	Helice crab		85.46	[20]
Tianjin	2015	Crab		341	[19]
Tianjin	2015	Mantis shrimp		45.5	[19]
Tianjin	2015	Shrimp		42	[19]
Tianjin	2015	Crab	48.0–428		[21]
Tianjin	2015	Shrimps	39.5–250		[21]
Hong Kong	2012	Portunus pelagicus		5.10 ± 0.77	[10]
Hong Kong	2012	Portunus sanguinolentus		31.29 ± 5.45	[10]
Hong Kong	2012	Metapenaeus ensis		9.77 ± 1.06	[10]
Hong Kong	2012	Parapenaeopsis tenella		9.00 ± 1.87	[10]
Hong Kong	2012	Harpiosquilla harpax		58.58 ± 4.51	[10]
Hong Kong	2012	Miyakea nepa		4.83 ± 1.78	[10]
		*Fishes*			
Dalian	2008	Large yellow croaker	3.4–8.7	5.2	[22]
Dalian	2008	Sliver pomfret	10.0–10.1	10.1	[22]
Tianjin	2011	Anchovy		141.9	[20]
Tianjin	2011	Octopus		263.91	[20]
Tianjin	2011	Weever		312.9	[20]
Tianjin	2011	Bartial flathead		378.2	[20]
Tianjin	2011	Sea catfish		989	[20]
Tianjin	2011	Haritail		229.9	[20]
Tianjin	2015	Mullet		2970	[19]
Tianjin	2015	Flathead		536	[19]
Tianjin	2015	Perch		128	[19]
Tianjin	2015	Tongue sole		366	[19]
Tianjin	2015	Porgy		41.6	[19]
Tianjin	2015	Goby		156	[19]
Tianjin	2015	Fish	73.9–1241		[21]
Tianjin	2008	Sliver pomfret	4.7–5.8	5.2	[22]
Qingdao	2008	Large yellow croaer	3.1–7.8	5.4	[22]
Qingdao	2008	Sliver pomfret	4.3–7.3	5.3	[22]
Shanghai	2008	Large yellow croaer	5.2–5.9	5.6	[22]
Shanghai	2008	Sliver pomfret	1.0–2.1	1.6	[22]
Zhoushan	2008	Sliver pomfret	1.4–1.5	1.4	[22]
Wenzhou	2008	Large yellow croaer	0.62–5.4	2.2	[22]
Wenzhou	2008	Sliver pomfret	0.57–0.69	0.64	[22]
Fuzhou	2008	Large yellow croaer	3.4–4.9	4.3	[22]
Fuzhou	2008	Sliver pomfret	0.85–1.4	1	[22]
Quanzhou	2008	Large yellow croaer	2.2–8.0	5.3	[22]
Quanzhou	2008	Sliver pomfret	0.85–2.0	1.5	[22]
Xiamen	2008	Large yellow croaer	4.6–5.9	5.3	[22]
Xiamen	2008	Sliver pomfret	0.78–1.7	1.1	[22]
Zhangzhou	2013	Striped mullet	0.27–17.9	9.45	[23]
Zhangzhou	2013	Red eelgoby	nd–10.3	nd	[23]
Zhangzhou	2013	Blue-spotted mudskipper	nd–15.4	nd	[23]
Hong Kong	2012	Clupanodon thrissa		7.84 ± 0.36	[10]
Hong Kong	2012	Thryssa kammalensis		55.66 ± 14.13	[10]
Hong Kong	2012	Ostorhinchus fasciatus		16.31 ± 2.87	[10]
Hong Kong	2012	Callionymus curvicornis		3.01 ± 1.26	[10]
Hong Kong	2012	Trypauchen vagina		21.84 ± 5.13	[10]
Hong Kong	2012	Leiognathus brevirostris		52.59 ± 8.98	[10]
Hong Kong	2012	Polydactylus sextarius		10.26 ± 1.50	[10]
Hong Kong	2012	Priacanthus macracanthus		6.01 ± 0.46	[10]
Hong Kong	2012	Pennahia argentata		19.24 ± 7.00	[10]
Hong Kong	2012	Johnius heterolepis		11.78 ± 0.73	[10]
Hong Kong	2012	Dendrophysa russelii		4.43 ± 1.67	[10]
Hong Kong	2012	Siganus canaliculatus		13.89 ± 3.86	[10]
Hong Kong	2012	Evynnis cardinalis		6.57 ± 2.87	[10]
Hong Kong	2012	Collichthys lucidus		43.02 ± 3.64	[10]
Hong Kong	2012	Trichiurus lepturus		15.88 ± 0.91	[10]
Hong Kong	2012	Cynoglossus arel		12.89 ± 2.07	[10]
Hong Kong	2012	Solea ovata		93.23 ± 10.73	[10]
Hong Kong	2012	Platycephalus indicus		29.12 ± 8.62	[10]
Hong Kong	2012	Inegocia japonica		12.22 ± 0.57	[10]
Zhuhai	2012	Striped mullet	nd–56.3	nd	[23]
		Mammals			
Hong Kong	1997–2007	Porpoises	4.1–501	55 ± 93	[24]
Hong Kong	1997–2007	Dolphin	32–519	168 ± 131	[24]
Hong Kong	2005–2015	Porpoises	97.2–6260		[25]
Hong Kong	2005–2015	Dolphin	447–45880		[25]

**Table 2:** Biomagnification factors (BMFs) and trophic magnification factors (TMFs) of HBCDs (lipid-weight basis) in marine food web from China and other countries.

Location	BMFs				TMFs				Reference
	ƩHBCDs	α-HBCD	β-HBCD	γ-HBCD	ƩHBCDs	α-HBCD	β-HBCD	γ-HBCD	
*China*									
Tianjin					1.68	1.74			[20]
Tianjin					1.72	1.75	1.83	1.64	[21]
Tianjin	0.9–28.1				5.6	10.8			[19]
Zhangzhou						0.17 *			[23]
Hong Kong	15.0–4160	16.8–7223	3.06–524	1.56–45.9	7.9	10.3			[10]
*Other Countries*									
The southern part of Korea					2.62	2–3	<1	2–3	[9]
Gdansk Zoo, Poland	5.6–8.3								[61]
Western Norway	0.5–7.9	2.8–26		0.03–2		2.6		0.3	[62]
Western Canadian Arctic		0.1–1.7							[63]
Eastern Canadian Arctic		<1–4		<1–17		2.1		0.5	[64]
King George Island, Antarctica					4.06	3.44	1.74	4.06	[7]

* Not significant (*p* > 0.05).

## Data Availability

Not applicable.
